# The Role of Physiotherapy in Chronic Osteomyelitis of the Second Metatarsal for the Restoration of Function in a 20-Year-Old Patient: A Case Report

**DOI:** 10.7759/cureus.54041

**Published:** 2024-02-11

**Authors:** Vaishnavi R Waghe, Subrat Samal

**Affiliations:** 1 Musculoskeletal Physiotherapy, Ravi Nair Physiotherapy College, Datta Meghe Institute of Higher Education & Research, Wardha, IND

**Keywords:** case report, pain, rehabilitation, chronic osteomyelitis, physiotherapy

## Abstract

Osteomyelitis, which is a bone inflammation brought on by an infectious agent, is a challenging clinical issue. Chronic osteomyelitis, characterized by persistent inflammation and infection of the bone tissue, poses significant challenges to both physical health and functional well-being. A 20-year-old male came with complaints of pus discharge from an ulcer present on the dorsum of the right foot with pain and swelling around it. He was unable to stand or walk properly so he came to Acharya Vinoba Bhave Rural Hospital, Wardha, India, where investigations were done which confirmed the diagnosis of chronic osteomyelitis of the second^ ^metatarsal. The patient then underwent debridement and curettage due to which he had trouble walking, his ankle joint's range of motion was restricted, and his ability to carry out everyday tasks was compromised. Physiotherapy rehabilitation was administered, and outcome measures were assessed, revealing notable enhancements in the patient's range of motion and muscular strength. A physiotherapy routine helped the patient overcome this, which is crucial to a quick and complete recovery. It also aided the patient's functional mobility and independent ambulation.

## Introduction

Osteomyelitis, an inflammatory bone condition triggered by infection, presents a multifaceted clinical challenge [1]. In underdeveloped areas, chronic osteomyelitis commonly evolves from neglected acute hematogenous osteomyelitis or traumatic incidents, such as wartime injuries. The condition manifests with regions of non-viable bone and soft tissue, termed sequestrum, serving as focal points for recurrent infections [2]. *Staphylococcus aureus* stands as the predominant causative pathogen for osteomyelitis in both developing and industrialized nations, accounting for approximately 70-80% of all identified microorganisms [3-5]. The pathogenesis of these diseases can manifest with acute, subacute, or chronic origins, influenced by various host and pathogen factors [6].

Chronic osteomyelitis, an uncommon autoimmune inflammatory disorder, predominantly affects children and young adolescents. It typically presents with persistent and severe bone pain at multiple sites, often accompanied by radiological evidence suggestive of osteomyelitis or neoplastic processes [7]. Osteomyelitis can affect any bone and exhibits diverse pathogenic mechanisms and clinical presentations. Surgical debridement is often imperative for the successful resolution of chronic or contiguous osteomyelitis [8]. Notably, osteomyelitis frequently arises as a complication of traumatic open fractures, particularly those associated with significant soft tissue damage [9]. In some instances, the treatment regimen is so intensive that patients may face prolonged disability, and even after years of remission, osteomyelitis is prone to recurrence. Some consider the primary objective of osteomyelitis therapy to be the containment rather than the complete eradication of the condition [10].

Various surgical techniques have been employed, including the use of antibiotic-impregnated bone cement and muscle transplants, yet bone infections persist as a significant clinical challenge [11]. A critical determinant of the efficacy of osteosynthesis procedures is the mechanical integrity of the bone tissue, which directly impacts the stability of surgical fixators, pivotal elements in the therapeutic regimen [12].

Acute hematogenous osteomyelitis predominates in children due to their developing bones and abundant blood circulation. Long tubular bones are commonly involved, with metatarsals representing roughly 2% of reported cases [13]. Advances in diagnostic precision and the ability to characterize the infection have been achieved through the expanded availability of sensitive imaging techniques, including plain radiography and direct sampling of wound material for microbial culture and antibiotic sensitivity testing. Surgical intervention, involving the removal of diseased and necrotic tissue, often becomes necessary during osteomyelitis treatment and relies on the judicious use of appropriate antibiotics [14].

Physiotherapy has consistently emerged as a pivotal element in expediting recuperation subsequent to various surgical interventions. In the context of osteomyelitis, the integration of tailored early physiotherapeutic interventions aligned with the individualized requirements and health status of the patient has demonstrated considerable merit in augmenting recovery. The physiotherapeutic rehabilitation protocol primarily centers on preventing secondary complications such as post-operative pain, effusion, pressure sores, and undertaking chest prophylaxis. Furthermore, the protocol emphasizes enhancements in strength, range of motion, and the attainment of functional milestones, all of which collectively contribute to fostering a successful recuperative outcome for the patient [15].

## Case presentation

Patient information

**Figure 1:**
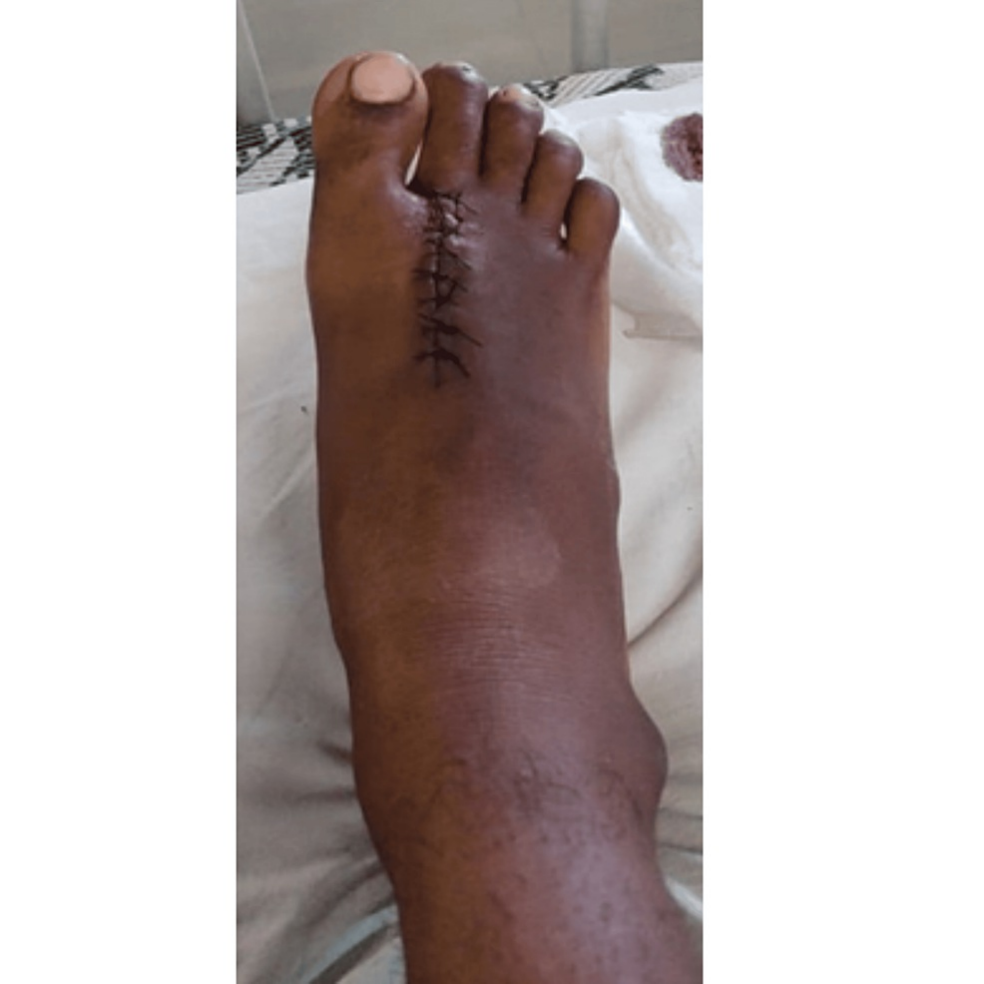
Right foot with suture over the dorsum side of the second metatarsal

A 20-year-old male presented with complaints of purulent discharge from an ulcer situated on the dorsum of his right foot on March 17, 2023. The patient reported being in good health approximately 15 years ago when he experienced a puncture injury from a thorn in his right foot, resulting in pain and localized swelling. In response, he sought care at a private hospital, where the thorn was removed and antiseptic measures were applied before being discharged. Approximately two to three years later, he once again experienced pain and the formation of pus at the site of the previous injury. This condition rendered him unable to stand or walk effectively, leading to his admission to a healthcare facility. At that time, a minor incision was made, and the accumulated pus was drained from the foot. Now, after a lapse of 12-13 years, the patient encountered a recurrence of pain and swelling at the previously infected site on his right foot, prompting his visit to Acharya Vinoba Bhave Rural Hospital for further evaluation. Diagnostic investigations were conducted, and the patient's pain was assessed using the Numerical Pain Rating Scale (NPRS), with a recorded score of 8/10 during movement and 5/10 at rest. The pain, localized to the dorsum of the right foot, was characterized as gradual in onset, with a dull, aching quality. It was exacerbated by physical activity and alleviated by rest and medications, with no diurnal variation noted (Figure 1).

Clinical findings

After obtaining the patient's consent, a comprehensive physical examination was performed while the patient was positioned supine lying. Vital signs were within normal limits. Upon local examination, the overlying skin exhibited a previous surgical scar measuring 2×0.5 cm in size, accompanied by swelling, localized warmth (elevated temperature), and grade 2 tenderness over the second metatarsal. There was also a diffuse swelling noted over the right foot. The physical examination encompassed manual muscle testing (MMT), revealing noteworthy findings. The patient exhibited 3 out of 5 strength in the right lower extremities for ankle plantarflexion and eversion, while 4 out of 5 strength was observed for dorsiflexion and inversion, due to the pain at the suture site over the dorsum side of the second metatarsal. Additionally, there was a limited range of motion, the specifics of which are provided in a table below.

Diagnostic assessment

**Figure 2:**
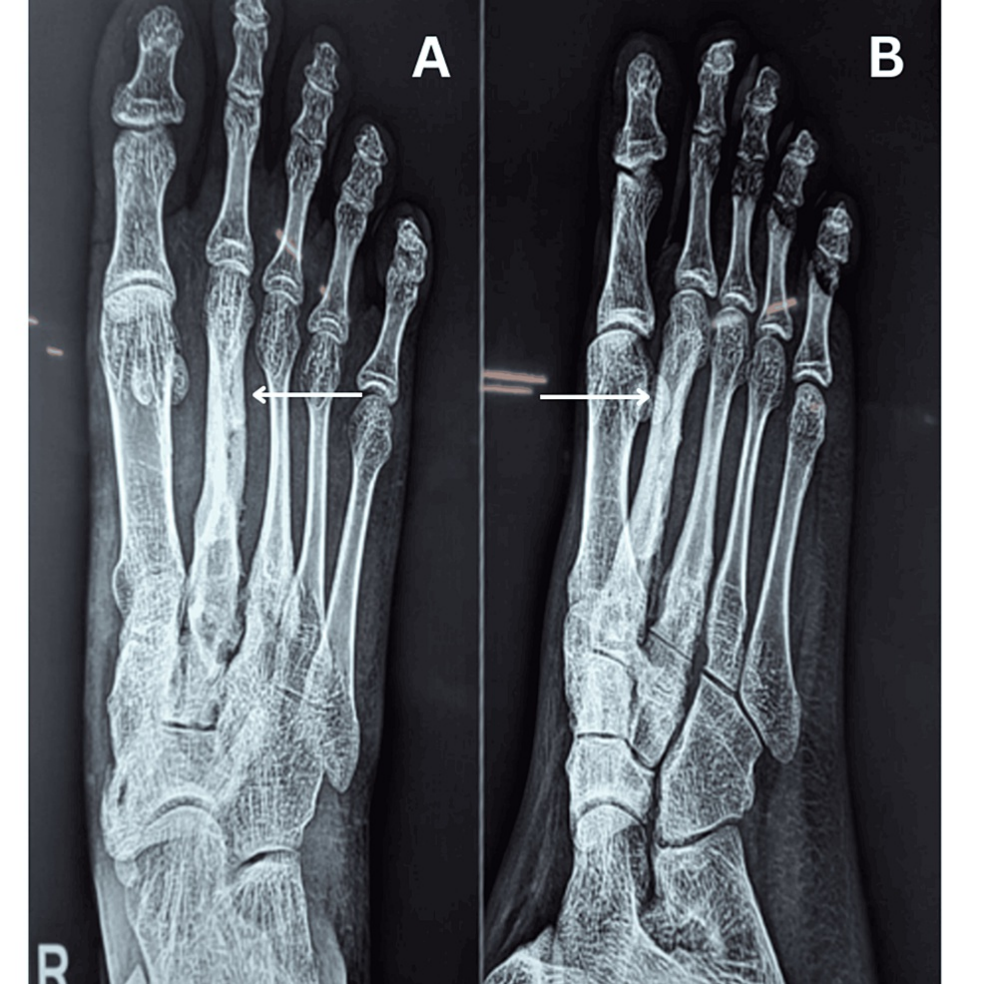
X-rays of the right foot before debridement of the second metatarsal: A) ankle AP view and B) ankle lateral view AP: anteroposterior

**Figure 3:**
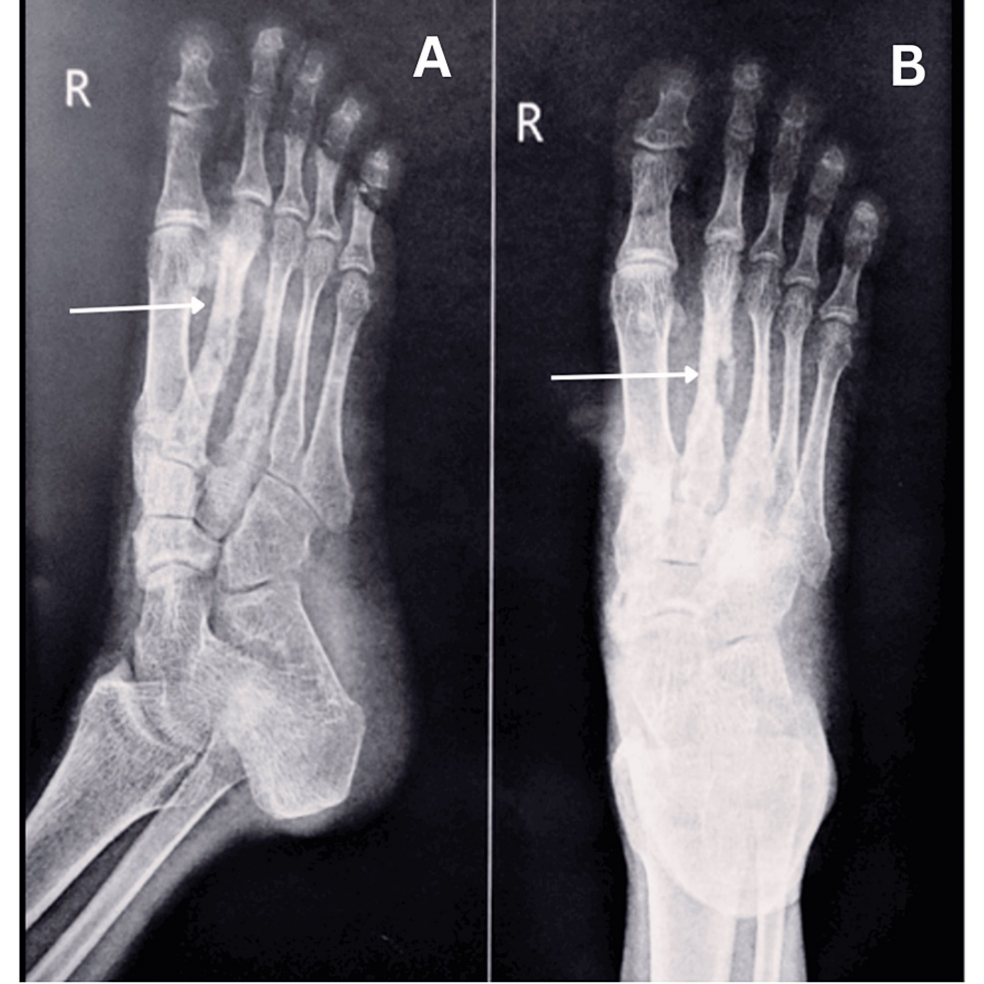
X-rays of the right foot after debridement and curettage of the second metatarsal: A) ankle lateral view and B) ankle AP view AP: anteroposterior

The patient underwent investigations like complete blood count, which showed normal white blood cell count and increased erythrocyte sedimentation rate (ESR) and C-reactive protein (CRP). Cytopathology shows cellular smears with multiple vascularized inflammatory cell fragments composed of proliferating endothelial cells surrounded by polymorphs, lymphocytes, and macrophages, and a few places show multinucleated histiocytic giant cells and macrophages with phagocytosis. Present cytomorphology is suggestive of "acute inflammatory exudative lesion with foreign body granuloma." Figure 2 shows the X-ray of the right foot before debridement of the second metatarsal, while Figure 3 shows the X-ray of the right foot after debridement and curettage of the second metatarsal.

Therapeutic intervention

**Figure 4:**
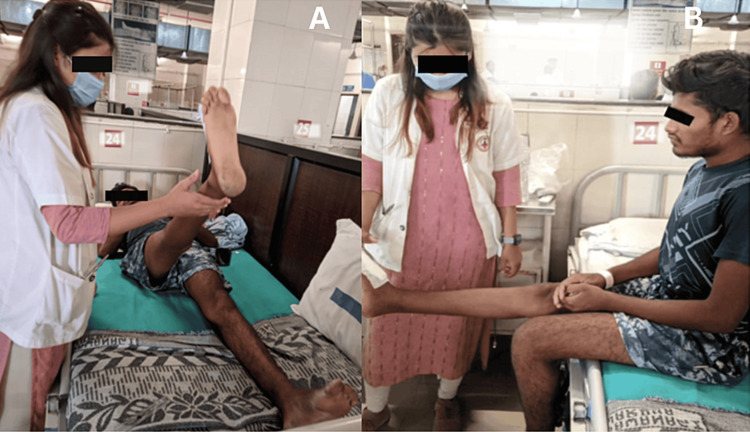
The patient is performing (A) straight leg raises with minimal assistance and (B) dynamic quadriceps

The short-term goals were patient education and to decrease pain on NPRS from 8/10 to 5/10 on activity and 5/10 to 3/10 on rest, restore functional range of motion, improve muscle strength and endurance of muscles of the foot, and prevent further complications, whereas the long-term goals were to decrease pain on NPRS from 5/10 to 3/10 on activity and 3/10 to 1/10 on rest, maintain muscle strength and endurance of muscles of the foot, encourage independent walking, and improve activities of daily living. The goal of the physical therapy rehabilitation regimen is to establish independent full weight-bearing walking without a walker and little assistance for activities of daily living by strengthening the left lower and both upper limbs while maintaining muscular integrity for the right lower limb. The phase-wise rehabilitation is shown in Table 1. Figure 4 shows the patient performing straight leg raises and dynamic quadriceps.

**Table 1:** Phase-wise rehabilitation protocol for eight weeks Reference: [16] UL: upper limb; LL: lower limb; NWB: non-weight bearing; TTWB: toe-touch weight bearing; PWB: partial weight bearing; FWB: full weight bearing; BD: twice a day; TD: thrice a day; reps: repetitions; secs: seconds; AAROM: active assisted range of motion; SLR: straight leg raise

Treatment	Week-wise dosage											
	Phase 1 (inpatient-day 1 to week 2)			Phase 2 (inpatient-up to week 4)			Phase 3 (outpatient-up to week 6)			Phase 4 (outpatient-up to week 8)		
Patient education: the patient should know what is going to happen to improve adherence	Patient education should be given in all phases of the protocol											
	Reps	Sets	Dose	Reps	Sets	Dose	Reps	Sets	Dose	Reps	Sets	Dose
Breathing exercises: to prevent hospital-acquired infections and to avoid pulmonary complications	10×10-second hold	1	BD-TD	10×10-second hold	2	BD-TD	-	-	-	-	-	-
Ankle-toe movements, bilateral	10	1	BD-TD	10	2	BD-TD	8-10	3	BD-TD	8-10	3	BD-TD
AAROM exercises for affected LL (ankle: inversion, eversion)	10	1	BD-TD	10	2	BD-TD	8-10	3	BD-TD	8-10	3	BD-TD
Heel slides, bilateral	10	1	BD-TD	10	2	BD-TD	8-10	3	BD-TD	8-10	3	BD-TD
SLR bilateral	10	1	BD-TD	10	2	BD-TD	8-10	3	BD-TD	8-10	3	BD-TD
Static quads and hams, bilateral	10	1	BD-TD	10×5-second hold	2×0.5 kg	BD-TD	8-10	2×1 kg	BD-TD	8-10	2×1.5 kg	BD-TD
Dynamic quads, bilateral	10×5-second hold	1	BD-TD	10×10-second hold	2	BD-TD	8-10	3	BD-TD	8-10	3	BD-TD
Strengthening of bilateral UL and LL with weight cuff	10×0.5 kg	1	BD-TD	10×0.5 kg	2	BD-TD	8-10	3	BD-TD	8-10	3	BD-TD
Sit to stand with a walker	10×10-second hold	1	BD-TD	10×10-second hold	2	BD-TD	8-10	3	BD-TD	8-10	3	BD-TD
Ambulation with walker	NWB on the right LL	1	BD-TD	TTWB on the right LL	2	BD-TD	PWB on the right LL	3	BD-TD	FWB on the right LL	3	BD-TD
Gait training exercises	-	-	-	-	-	-	10	1	BD-TD	10	2	BD-TD

Follow-up and outcomes

Furthermore, Table 2 shows the baseline and post-physiotherapy muscle strength measurements of the lower limb joints. The beginning NPRS score was 8/10, but by the time the patient was discharged, it had dropped to 2/10.

**Table 2:** Muscle strength evaluation of the right foot 3: full ROM against gravity; 4: full ROM against gravity, moderate resistance; 5: full ROM against gravity, maximal resistance ROM: range of motion

Movements at the ankle	Baseline	Discharge
Plantarflexors	3/5	4/5
Dorsiflexors	4/5	5/5
Invertors	4/5	5/5
Evertors	3/5	4/5

The active and passive range of motion noted during the first evaluation and at the end of the physiotherapy session is shown in Table 3.

**Table 3:** AROM and PROM testing of the right foot AROM: active range of motion; PROM: passive range of motion

Movements	Baseline		Discharge	
	AROM		PROM	
Plantarflexion	0-20°	0-22°	0-30°	0-35°
Dorsiflexion	0-15°	0-17°	0-18°	0-20°
Inversion	0-25°	0-27°	0-32°	0-35°
Eversion	0-5°	0-8°	0-10°	0-12°

## Discussion

Chronic osteomyelitis manifests frequently among pediatric populations residing in rural regions, notably in countries like India [17]. Within India and other economically disadvantaged nations, chronic osteomyelitis of the extremities remains a significant etiology of morbidity and functional limitations in children [18]. Although commonly associated with conditions such as sickle cell disease, the patient under consideration did not present with any concurrent medical comorbidities. Rather, the patient's medical history disclosed a thorn injury that had not been completely excised and had persisted embedded for a minimum of two years. Consequently, the patient underwent a minor surgical intervention for pus drainage from the foot, potentially contributing to the onset of chronic osteomyelitis. Studies suggest that osteomyelitis affects an estimated 2.9 per 100,000 children within the pediatric demographic [19]. The incidence of osteomyelitis subsequent to foot puncture can escalate up to 16% in the general populace and even higher (30-40%) among individuals afflicted with diabetes [20].

The management of chronic osteomyelitis demands a comprehensive approach, necessitating the collaboration of skilled orthopedic physiotherapists [17]. Physical therapy assumes a pivotal role in the treatment regimen for chronic osteomyelitis, facilitating enhanced ambulation and functional autonomy, thus expediting the recuperative trajectory. In the instance under review, the patient's mobility and daily activities were impeded by discomfort, necessitating the judicious use of analgesic agents for pain relief. Given the patient's ambulatory challenges, our principal objective was to foster autonomous full weight bearing and proficient engagement in daily tasks with minimal support.

The outcomes of the case study underscore the effectiveness of a multidisciplinary approach, integrating definitive surgical interventions with tailored physiotherapeutic rehabilitation, resulting in significant improvements in the patient's functional autonomy and ambulatory prowess [16]. These advancements notably accelerate the recovery process, facilitating swift and favorable rehabilitation outcomes. Prolonged management of chronic osteomyelitis may substantially impair ambulation, functional mobility, and independence, as evidenced in this and similar cases [15]. However, in our investigation, the initiation of physiotherapy proved instrumental in addressing these impediments, playing a pivotal role in enhancing the patient's functional independence.

It is noteworthy that physiotherapy has not been consistently incorporated into interdisciplinary strategies for managing chronic osteomyelitis in many case studies, potentially prolonging healing durations. In our case study, rehabilitation objectives were meticulously devised to augment the patient's daily activities and facilitate supervised full weight-bearing ambulation. This study underscores the critical role of physiotherapy in ameliorating functional mobility and ambulation among chronic osteomyelitis patients undergoing debridement and curettage, a therapeutic approach that is relatively underutilized but may confer significant benefits, particularly in individuals with diabetic foot complications.

## Conclusions

Chronic osteomyelitis frequently afflicts children residing in rural areas, particularly in regions like India. In this case, physiotherapy plays a pivotal role in the management of chronic osteomyelitis affecting the second metatarsal in a 20-year-old male. Utilizing a multifaceted approach integrating therapeutic exercises, manual techniques, and patient education, the physiotherapeutic intervention demonstrates substantial advancements, notably in pain management, augmentation of range of motion, enhancement of muscle strength, improved gait, and prevention of secondary impairments. This eventually allowed for a secure return to daily activities.
